# Pleomorphic Carcinoma of the Breast: A Report of Three Cases

**DOI:** 10.7759/cureus.61091

**Published:** 2024-05-26

**Authors:** El Achchi Anass, Majdoubi Amine, El Hammouti Mohamed, Tariq Bouhout, Badr Serji

**Affiliations:** 1 Surgical Oncology Department, Regional Oncology Center, Mohammed VI University Hospital, Oujda, MAR; 2 Faculty of Medicine and Pharmacy, Mohammed First University, Oujda, MAR

**Keywords:** systemic chemotherapy, mastectomy, giant cell, pleomorphic carcinoma, breast carcinoma

## Abstract

Pleomorphic carcinoma (PC) is an uncommon and high-grade form of breast carcinoma characterized by the presence of distinctive pleomorphic giant tumor cells exhibiting bizarre nuclei and atypical mitosis.

In this study, we report three patients who presented with lesions composed of a proliferation of large pleomorphic cells with a predominance of multinucleated giant cells on a microscope. Immunohistochemical analysis revealed distinct immunologic profiles within the respective malignant components. Notably, this report aims to contribute valuable insights, adding to the understanding of this uncommon tumor, accompanied by a literature review.

Despite its rarity, PC in the breast remains clinically relevant due to its distinctive morphological and pathological features. These unique attributes require specific considerations in both clinical presentation and management.

## Introduction

Breast cancer ranks as the most prevalent cancer for women, causing 670,000 deaths worldwide each year [1], and encompasses various pathologic subtypes, leading to significantly diverse prognoses and management approaches [1]. Invasive lobular carcinoma (ILC) of the breast ranks as the second most prevalent form of invasive breast carcinoma, representing 8% to 14% of all breast cancer cases [2]. Pleomorphic carcinoma (PC) is an uncommon and high-grade form of breast carcinoma characterized by the presence of distinctive pleomorphic tumor cells with unusually large and irregular nuclei, along with atypical mitosis. Initially, as outlined by Page and Anderson [3] in 1987 and identified by Dixon et al. [4] as a hybrid subgroup of invasive lobular carcinoma, it was later distinguished from other cancers due to its aggressive behavior and its association with worse outcomes than other subtypes of breast cancer [5].

According to the 2012 World Health Organization (WHO) classification [6], pleomorphic carcinoma is identified as a rare variant of high-grade invasive carcinoma of no special type of breast. It is characterized by the proliferation of pleomorphic and irregular tumor giant cells, constituting more than 50% of the tumor cells, within a backdrop of adenocarcinoma or adenocarcinoma with spindle or squamous differentiation.

## Case presentation

Patient 1

**Figure 1:**
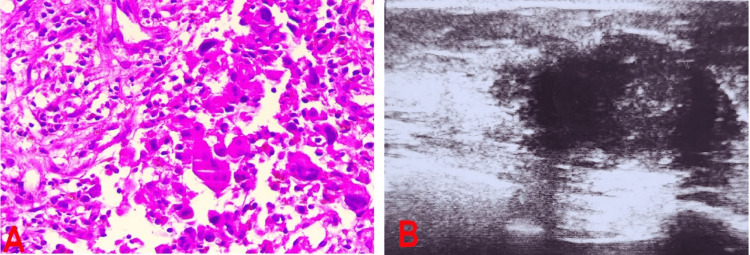
Test results A: Histological image showing pleomorphic breast carcinoma; B: Ultrasonography revealed an infiltrative, irregularly shaped, and poorly circumscribed hypoechoic mass measuring 30×21 mm, which was classified as BI-RADS IV.

A 52-year-old woman with no medical history discovered a mass in her right breast and sought consultation at our department two months later. Physical examination revealed a painless and firm mass in the upper outer quadrant of the right breast measuring approximately 3 cm in diameter, accompanied by inflammatory signs, and a single enlarged lymph node was palpated in the right axilla. On the left breast, no anomalies were observed. Ultrasonography and mammography revealed an infiltrative, irregularly shaped, and poorly circumscribed hypoechoic mass measuring 30×21 mm, which was classified as BI-RADS IV (Figure 1B). The pathological results of a core needle biopsy revealed a PC with no hormone receptor expression (Figure 1A). Subsequent staging assessments revealed no secondary lesions. The patient underwent neoadjuvant chemotherapy based on the EC100 protocol (six cycles), followed by radical mastectomy and node dissection of the right axillary region. The pathological stage was pT2N1M0, indicating four cycles of adjuvant chemotherapy. After six months of follow-up, the patient remained alive with no signs of cancer recurrence or metastasis.

Patient 2

**Figure 2:**
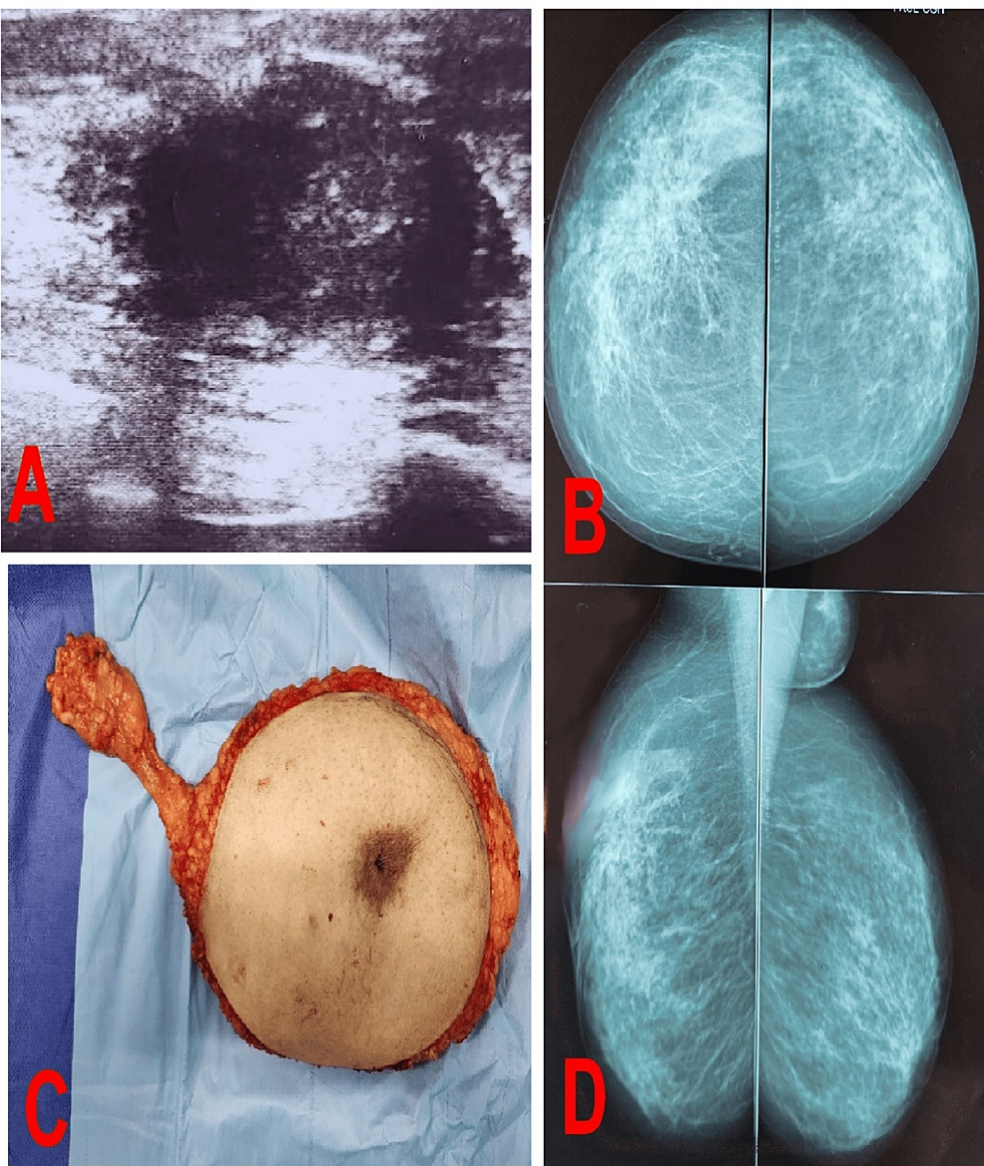
Findings A: Ultrasonography revealed a BI-RADS V lesion with speculated contours that was moderately hypoechoic with posterior shadowing, measuring 50x25 mm in its large axes; B, D: Mammography revealed a BI-RADS V measuring 50x25 mm in its large axes; C: Surgical specimen of a right mastectomy with axillary clearance.

A 41-year-old woman had a mass in her right breast. Upon admission, the examination revealed a hard mass measuring 6 cm in the upper quadrants of the right breast, and one swollen lymph node was palpated in the right axilla. There were no inflammatory signs or abnormalities in the left breast or secondary lesions at the staging assessment. Ultrasonography-mammography revealed a BI-RADS V lesion with speculated contours that were moderately hypoechoic with posterior shadowing, measuring 50x25 mm in its large axes (Figure 2A, 2B, 2D). The pathological results of the core needle biopsy were in favor of a PC. Total mastectomy and axillary lymphadenectomy of the right breast were performed (Figure 2C), and the patient was pathologically confirmed.

Patient 3

**Figure 3:**
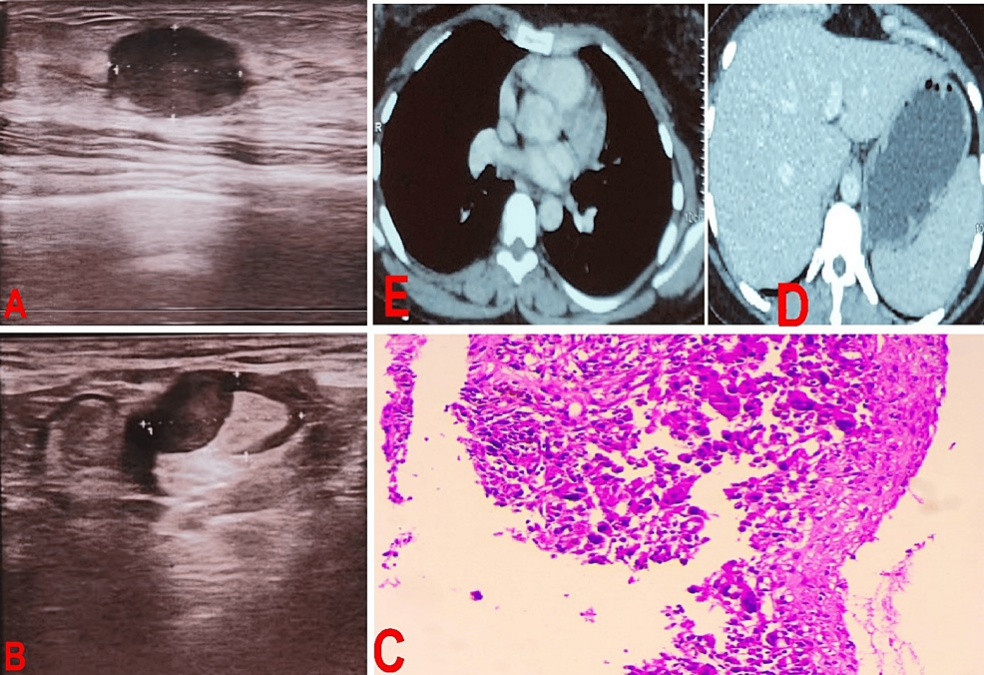
Test results A, B: Ultrasound mammography indicated an infiltrative, irregularly shaped hypoechoic mass measuring 50x30 mm in size associated with multiple lymph nodes, and the lesion was categorized as a BI-RADS V; C: High-magnification histological image showing cellular details; D, E: CT scan image reveals no secondary lesions.

A 49-year-old woman discovered a nodule in the superior outer quadrant of her right breast. She was referred to our department after consulting a gynecologist, and ultrasound mammography indicated an infiltrative, irregularly shaped hypoechoic mass measuring 50x30 mm in size associated with multiple lymph nodes. The lesion was categorized as BI-RADS V (Figures 3A, 3B). Pathological examination of a core needle biopsy revealed a PC (Figure 3C). No secondary lesions were found on the CT scan (Figures 3D, 3E), and the patient underwent neoadjuvant chemotherapy based on concomitant chemoradiotherapy, followed by radical mastectomy with axillary lymph node dissection (T4N3aMx) and four cycles of adjuvant chemotherapy. The patient was still alive and had no signs of cancer recurrence or metastasis.

## Discussion

PC is a subtype of breast tumor that exhibits distinctive morphological characteristics. It is primarily composed of pleomorphic cells, which constitute more than 50% of the tumor cells, and many of them are large and multinucleated. The initial report on PC provided by Silver et al. [7] suggested that it represents the morphological spectrum of Grade III invasive ductal carcinoma (IDC), and according to the recent WHO classification, this tumor has been categorized as a subtype of invasive carcinoma (nonspecial type). Due to its relatively infrequent occurrence, there is a lack of evidence to establish specific protocols for its treatment. Similar to all types of breast cancer, surgical resection remains the cornerstone of treatment. Existing datasets on PC indicate a tendency for larger tumors at the time of diagnosis, coupled with higher-grade characteristics and an increase in lymph node involvement [8]. As a result, a more aggressive surgical approach can be anticipated, with a higher probability of mastectomy being necessary for patients with PC [8]. In one study, the mastectomy rate was reported to be 63.5% for PCs, whereas it was 28.8% for IDCs and 38.6% for ILCs [9]. While it is widely acknowledged that adjuvant chemotherapy provides less advantage in ILC when contrasted with IDC, there is no evidence to support this assertion for PC. Some oncologists lean toward offering adjuvant chemotherapy for PC, influenced partly by the unfavorable pathological and biomarker characteristics at diagnosis, along with the belief that PLC leads to poorer outcomes. In the study by Monhollen et al., 23 out of 24 patients diagnosed with Stage II to III disease received adjuvant chemotherapy [10]. The use of adjuvant trastuzumab and hormonal therapy depends on tumor markers rather than pleomorphic histology. In the same study, two patients with human epidermal growth factor receptor 2 (HER-2) positivity who received trastuzumab showed varying results, with one experiencing progressive disease at 27 months and the other achieving a disease-free survival of > 60 months. Similarly, limited information is available regarding the use of neoadjuvant chemotherapy in locally advanced cancers that are PLCs, and classic ILC patients exhibit a lower complete response rate with neoadjuvant chemotherapy than invasive ductal carcinoma patients. PC was not defined or treated as a separate entity [11]. In one study focusing on neoadjuvant chemotherapy combined with ILC, only seven patients with locally advanced PC were included. Within two years of surgery, four patients (57%) developed distant metastases, and 71% of PLCs exhibited substantial residual disease [12]. Notably, a substantial proportion (five out of seven patients) of patients were residual cancer burden class III after chemotherapy, indicating a poor response of PC tumors to neoadjuvant chemotherapy. PC is disproportionately prevalent among metastatic breast cancers [10]. The approach to managing metastatic disease relies on extrapolating data from other types of breast cancer. Significantly, a limited case series involving four patients reported an exceptionally high sensitivity of metastatic PCs to trastuzumab [13]. In terms of prognosis, PC is an aggressive variant of ILC [14]. Larger tumors, advanced-grade disease, elevated lymph node positivity, increased HER-2 expression, and a potentially heightened incidence of triple-negative breast cancer (TNBC) have been identified, as outlined earlier. Each of these factors represents a negative prognostic indicator for breast cancer patients.

Eusebi et al. [5] reported 10 cases in 1992, revealing that 60% of patients passed away within 42 months and 90% suffered relapse within two years. This rare yet highly aggressive tumor is a cause for concern. Subsequent publications have consistently indicated that PLC tumors have worse outcomes compared to classic ILC [14]. Furthermore, patients with PC have a greater risk of metastasis and recurrence than patients with other invasive carcinomas. Recent publications have reevaluated patient outcomes in PC, attributing poorer prognoses less to the pleomorphic histological subtype and being more inclined to be associated with negative prognostic factors [15]. In our study, despite positive lymph node metastasis, the patients did not experience recurrence or metastasis six months after the operation. Table 1 is a comparative table with other studies.

**Table 1:** Comparative table with other studies reported in the literature *  Number of cases with positive lymph nodes

Author	Reference	Year	Cases	Age (year)	Tumor size (cm)	Neoadjuvant therapy	Surgery	Lymph nodes status	Adjuvant therapy	Survival (months)
Our study	-	2022	3	41-52 (median,46.5)	3-5 cm (median,4cm)	2/3	Mastectomy	3/3 positive*	3/3	No recurrence
Peña-Jaimes L et al.	[16]	2018	1	72	2,3 cm	No	Mastectomy	Positive	Yes	24
Alicia et al.	[17]	2012	2	46-76 (median, 61)	1-5 cm (median, 2.5 cm)	Not determined	Mastectomy/Segmental resection	1/2 positive*	Not determined	Not determined
Christopher V et al.	[18]	2010	37	23-78 (median, 50,5)	Not determined	12/37	Mastectomy/Breast-conserving surgery	17/37 positive*	18/37	0,1-78
Jing Zhao et al.	[19]	2010	10	33-76 (median, 54,5)	1-15 cm (median, 8cm)	Not determined	Mastectomy/Breast-conserving surgery	4/10 positive*	Not determined	27-81
Rin Y et al. (25)	[20]	2010	1	17	7,6 cm	No	Mastectomy	Positive	Yes	17
Silver SA	[7]	1999	16	30-96 (median, 63)	0.5-15 cm (median, 7.75cm)	No	Mastectomy/Lumpectomy/Modified radical mastectomy	12/16 positive*	Yes	0-110

## Conclusions

Despite its rarity, PC in the breast remains clinically relevant due to its distinctive morphological and pathological features. These unique attributes require specific considerations in both clinical presentation and management. Recognized as a more aggressive subtype of invasive lobular carcinoma, PC underscores the importance of further research to refine our understanding of its clinical behavior and to develop optimal treatment recommendations.
